# Long Non-coding RNA LINC02474 Affects Metastasis and Apoptosis of Colorectal Cancer by Inhibiting the Expression of GZMB

**DOI:** 10.3389/fonc.2021.651796

**Published:** 2021-04-09

**Authors:** Tiantian Du, Qinglun Gao, Yinghui Zhao, Jie Gao, Juan Li, Lili Wang, Peilong Li, Yunshan Wang, Lutao Du, Chuanxin Wang

**Affiliations:** ^1^ Department of Clinical Laboratory, The Second Hospital, Cheeloo College of Medicine, Shandong University, Jinan, China; ^2^ Department of Hepatobiliary Surgery, Shandong Provincial Third Hospital, Cheeloo College of Medicine, Shandong University, Jinan, China; ^3^ Department of Clinical Laboratory, Qilu Hospital of Shandong University, Jinan, China; ^4^ Shandong Engineering & Technology Research Center for Tumor Marker Detection, The Second Hospital of Shandong University, Jinan, China; ^5^ Shandong Provincial Clinical Medicine Research Center for Clinical Laboratory, The Second Hospital of Shandong University, Jinan, China

**Keywords:** long non-coding RNA, colorectal cancer, migration, invasion, apoptosis

## Abstract

**Background:**

Colorectal cancer (CRC) is one of the most frequently diagnosed malignancies. Metastasis is the main event that impedes the therapeutic effect on CRC, and its underlying mechanisms remain largely unclear. LINC02474 is a novel long noncoding RNA (lncRNA) associated with metastasis of CRC, while little is known about how LINC02474 regulates these malignant characteristics.

**Methods:**

Expressions of LINC02474 and granzyme B (GZMB) were assessed by quantitative real-time polymerase chain reaction (qRT-PCR) or Western blotting analysis. Cell metastasis was detected by transwell assay and metastatic nude mouse model, and apoptosis was determined by Western blotting analysis and flow cytometry. Besides, the interaction between LINC02474 and GZMB was detected by dual-luciferase reporter assays.

**Results:**

The expression of LINC02474 was significantly up-regulated in CRC tissues. Moreover, depletion of LINC02474 damaged the metastatic abilities of CRC cells *in vivo and in vitro* while boosting apoptosis. Besides, up-regulation of LINC02474 could promote migration and invasion, while apoptosis was inhibited in CRC cells. Besides, down-regulation of LINC02474 promoted the expression of GZMB, and interference of GZMB could increase the metastatic abilities of CRC cells while reducing apoptosis. Furthermore, LINC02474 was related to the transcriptional repression of GZMB in CRC cells determined by the dual-luciferase reporter assay.

**Conclusions:**

The findings revealed that a novel lncRNA, LINC02474, as an oncogene, could promote metastasis, but limit apoptosis partly by impeding GZMB expression in CRC. Besides, LINC02474 had the potential to be used as a biomarker in the prognosis of CRC.

## Introduction

The latest epidemiological investigation has shown that colorectal cancer (CRC) ranks as the third most common diseases of cancer-related deaths in the Western countries among both man and woman. It is considered to be the second among the general population. Even with advances in the screenings and therapeutic strategies of CRC in recent years, it is estimated that 53,200 CRC-related deaths will occur in 2020 (1, 2). The leading cause of patient death at advanced stages is metastasis of CRC cells to tissues and organs outside where the tumor developed, leading to increased overall mortality (3). Almost a quarter of CRC patients at an advanced level have metastasis at diagnosis, as there are sometimes no signs at the early stage of CRC before it enters the advanced stage (1). Current treatment regimens can only relieve CRC patients’ symptoms at an advanced stage, 5-year survival rate of advanced CRC is only 10% to 20% (4–6). Therefore, it is urgently necessary to explore neo-biomarkers for CRC and understand their pathological mechanism to improve patients’ survival with advanced CRC (7, 8).

Current evidence indicates that non-coding RNAs (ncRNAs) occupy more than 70% of the human genome (9). Among ncRNAs, those with a nucleotide chain longer than 200 are regarded as long non-coding RNAs (lncRNAs) (10). LncRNAs are attract increasing attention due to their influential regulatory roles in multiple biological processes, especially oncogenesis and cancer pathogenesis (11–16). Moreover, functional dysregulation of some lncRNAs affects cancer cells’ progression and death (17–20). In facts, some researches have reported various dysregulated lncRNAs in CRC. For instance, RAMS11 have been reported associated with poor survival and aggressive phenotypes (21), and overexpression of CCAT2 promotes chromosomal instability (CIN) of CRC cells (22). Also, given the cancer-related characteristics of lncRNAs, some of the lncRNAs may serve as potential biomarkers in CRC.

Granzyme B (GZMB) is generally produced in the tumor microenvironment by cytotoxic T lymphocytes (CTLs) and natural killer (NK) cells as a toxic granule-secreted enzyme with killing activity (23–25). However, a few reports have recorded that low GZMB expression could be associated with early CRC metastasis (26). Moreover, as a serine protease, GZMB has hydrolytic activity and can cleave downstream caspase-3 and Bid to activate them, contributing to apoptosis of targeted cells (27, 28).

Here, we aimed to explore the possible biological function of a novel lncRNA, LINC02474, in the progression and metastasis of CRC. LINC02474 was highly expressed among cancerous areas. Moreover, depletion of LINC02474 inhibited the metastatic ability of CRC cells but intensified the apoptosis. Besides, LINC02474 might exert its effect by mediating transcriptional dysregulation of GZMB. Altogether, our current findings provided significant insights into the regulatory role of LINC02474 in the progression and metastasis of CRC.

## Materials and Methods

### Human Specimens and Ethics Statement

A total of 80 paired CRC and adjacent non-tumor tissue specimens were obtained from CRC patients who underwent a surgical operation during 2016 and 2018 at the Second Hospital of Shandong University. All enrolled patients neither received preoperative chemotherapy or radiotherapy nor had other cancer at the specimen collection time. Histopathological grades were staged based on the 8th edition of the Cancer Staging Manual of the American Joint Committee on Cancer (AJCC). All collected tissue specimens were immediately snap-frozen in liquid nitrogen and stored at −80°C. The essential demographic characteristics and clinical information of these 80 CRC patients were obtained from medical records. All tissue specimens were collected in compliance with the informed consent policy, and this experiment was approved by the Committee for Ethical Review of Research involving Human Subjects of the Second Hospital, Cheeloo College of Medicine, Shandong University.

### Cell Culture

The human CRC cell lines (DLD-1, SW480, HT-29, HCT116, SW1116, and LOVO), one standard colorectal epithelial cell line (FHC), and HEK293T cells were purchased from the cell bank of the Chinese Academy of Sciences (Shanghai, China). DLD-1, SW480, HT-29, HCT116, SW1116, FHC, and HEK293T cells were maintained in Dulbecco-modified essential medium (DMEM) (Gibco, Shanghai, China, Cat#11995500BT). Meanwhile, HCT116 cells were cultured in RPMI-1640 medium (Gibco, Shanghai, China, Cat#C11875500BT). All culture media contained 1% penicillin and streptomycin (Solarbio, Beijing, China, Cat#P1400) and 10% fetal bovine serum (FBS) (Sagecreation, Beijing, China). All cells were maintained at 37°C with 5% CO2, tested negative for mycoplasma contamination, and authenticated based on STR fingerprinting before use.

### SiRNA, Plasmid Construction, and Cell Transfection

Three individual siRNAs specific for LINC02474 (si-LINC02474 1#, 2#, and 3#) and GZMB (si-GZMB 1#, 2#, and 3#), as well as a scrambled negative control siRNA, were acquired from GenePharma (Shanghai, China). Full-length cDNA of human LINC02474 (441bp) was synthesized and cloned into the pcDNA3.1 plasmid vector (Obio Technology, Shanghai, China). The shRNA of LINC02474 was synthesized and cloned into the pLKD-shRNA plasmid vector (GeneCreat, China). All plasmid vectors were extracted by the Endo-Free Plasmid Mini Kit (Omega Bio-Tek, USA, Cat#D6950). The siRNAs or plasmid vectors were transfected into cells with Lipofectamine 2000 (Invitrogen, Cat#11668019) and OPTI-MEM (Gibco, Shanghai, China, Cat#31985062) mixture after a 20 min incubation following the manufacturer’s instructions.

To establish stable LINC02474 depletion or overexpression cells, three-plasmid lentiviral packaging systems (plasmid vectors specific for LINC02474, PAX, and pMD2G) and HEK293T cells were used to produce supernatant containing viral particles after more than 48 h of cultivation. Then the supernatant was collected and incubated with the CRC cells for at least 48 h, and the positive cells were then selected by puromycin (2.5 μg/ml) (Solarbio, Beijing, China, Cat#P8230). The expression efficiency was examined by fluorescence microscope and qRT-PCR. All sequences of siRNA and shRNA are listed in **Supplementary Table 2**.

### RNA Extraction and qRT-PCR Analysis

Total RNA was collected from tissues with TRIzol Reagent (Ambion, Invitrogen, Carlsbad, CA, USA, Cat#10296010), Simultaneously, it was purified from cultured cells by RNA fast 2000 Reagent (Fastagen, Shanghai, China, Cat#220011). Purified RNA was quantified with a NanoDrop spectrophotometer 2000 (Thermo Fisher Scientific, Waltham, MA, USA) and then reversely transcribed into cDNA using random primers with the PrimeScript™ RT Reagent Kit (TaKara, Dalian, China, Cat#RR037A). qRT-PCR was processed with TB Green™ Premix Ex Taq™ (TaKara, Dalian, China, Cat#RR420A) on a CFX-96 real-time PCR System (Bio-Rad, Shanghai, China). Briefly, after an initial denaturation at 95°C for 30 s, the amplifications were carried out with 42 cycles at a melting temperature of 95°C for 5 s and an annealing temperature at 58°C for 30 s. Glyceraldehyde-3-phosphate dehydrogenase (GAPDH) was served as an endogenous control. The 2^-ΔΔCT^ method was used to calculate the relative expressions of target genes. The specific primers used for qRT-PCR are listed in **Supplementary Table 3**.

### Western Blotting Analysis and Antibodies

Cells were washed with phosphate-buffered saline (PBS) and then lysed with a Western/IP lysis buffer (Beyotime, Shanghai, China, Cat#P0013) contained a protease inhibitor cocktail (Roche Applied Science, Indianapolis, IN, USA). After 30 min of lysis on ice, the whole lysates were centrifuged at 12,000 rpm for 30 min at 4°C. The proteins were then quantified with a bicinchoninic acid protein assay kit (Vazyme Biotech Co., Ltd., Nanjing, China, Cat#E112-01/02) after a 30 min incubation at 37°C. Subsequently, proteins were subjected to sodium dodecyl sulphate-polyacrylamide gel electrophoresis (SDS-PAGE) on 10% or 12% gels and electrotransferred onto 0.22-μm polyvinylidene difluoride membranes (Millipore, USA, Cat# SLGVR33RS). Then, the membranes were blocked with 5% bovine serum albumin (BSA) (Solarbio, Beijing, China, Cat#A8020) and 1% Tween-20 (Solarbio, Beijing, China, Cat#T8220) in PBS at room temperature for 1.5 to 2 h, followed by incubation with primary antibodies against GAPDH (CST, #5174S, 1:2,000), β-actin (CST, #8457, 1:2,000), GZMB (CST, #17215, 1:1,000), cleaved caspase substrate (CST, #8698, 1:1,000), cleaved caspase-3 (CST, #9664, 1:1,000), cleaved PARP (CST, #5625, 1:1,000), caspase-3 (CST, #9662, 1:1,000), PARP (CST, #9532, 1:1,000), and Bid (CST, #2002, 1:1,000) at 4°C overnight. Tris-buffered saline containing 1% Tween-20 was used to wash the membranes (four times, 5 min for each), followed by incubation with horseradish peroxidase-conjugated secondary antibodies (at a dilution of 1:2,000) at room temperature for 1 h. Next, the bands were visualized with the high-sensitivity ECL Chemiluminescence Detection Kit (Vazyme Biotech Co., Ltd., Nanjing, China, Cat#E412-01-AA) and an enhanced chemiluminescence analysis system (Bio-Rad, Shanghai, China). GAPDH and β-actin were used as controls. All images were quantified by ImageJ software. The assay was repeated at least three times.

### Cell Migration and Invasion Assays

Cell migration and invasion assays were performed with 24-well transwell chambers (8-μm pore size, Corning). Briefly, for migration assays, single-cell suspension at appropriate densities (8 × 10^4^ cells/per well for DLD-1, SW1116, and SW480 cells or 1 × 10^5^ cells/per well for LOVO cells) was plated into the upper chamber with 300 μl serum-free medium. In comparison, the lower section was filled with a 600 μl medium containing 20% FBS. After 24 h (DLD-1, SW1116, and SW480) or 48 h (LOVO), the chamber was washed with PBS. Then cells trapped in the upper chamber were removed, and sections in the lower surface were fixed with methanol and stained with crystal violet (0.1%) (Solarbio, Beijing, China, Cat#G1064). The cell numbers were determined using a microscope from five randomly selected visual fields. For invasion assays, similar methods were performed except that the upper chamber was pre-coated with Matrigel (BD Biosciences, San Jose, CA, United States, Cat# 356234), the cell numbers were doubled, and the collection time was prolonged to 48 h for DLD-1, SW1116, and SW480 cells and 72 h for LOVO cells. These assays were repeated at least three times.

### Flow Cytometry Analysis

The cells were digested with trypsin digestion solutions without EDTA (0.25%) (Solarbio, Beijing, China, Cat#T1350) and harvested. Centrifuging 5 min with 350g. The collected cells were washed with PBS two times, then stained with Annexin V-APC/propidium iodide (PI) apoptosis detection kit (BestBio, China, Cat#BB-41033) following the manual instruction. And finally analyzed with flow cytometry (BD Biosciences) according to the manufacturer’s instructions. The assay was repeated at least three times.

### RNA FISH

Fluorescence processed RNA FISH assay in Situ Hybridization Kit (GenePharma, Shanghai, China). Cy3-labeled LINC02474 and β-actin sense probe and Cy3-labeled LINC02474 antisense probe were acquired from RiboBio (China). DLD-1 cells were first fixed with 4% formaldehyde (Biosharp, Cat#BL539A) for 15 min and then permeabilized in PBS containing 0.1% Triton X-100 (Solarbio, Beijing, China, Cat#T8200) at room temperature for 15 min. After incubated with 2× SSC at 37 °C for 30 min, the cells were hybridized with labeled FISH probe pre-mixed solution at 37 °C overnight in the dark. Subsequently, the cells were washed with 0.1% Tween-20 for 5 min and then sequentially washed with 2× SSC and 1× SSC for 5 min. The three reagents mentioned above were warmed up to 42°C before use. Finally, 4,6-diamidino-2-phenylindole (DAPI) (Solarbio, Beijing, China, Cat#C0065) was used to stain the cell nucleus for 10 min. The images were produced by fluorescence microscopy (Carl Zeiss Microscopy, LLC, USA).

### RNA-seq Analysis

Total RNA was isolated from DLD-1 cells, in which LINC02474 was stably depleted, and their control cells using the RNeasy mini kit (Qiagen, Germany, Cat#74104). Then the cDNA library was acquired and validated by Agilent 2100 bioanalyzer (Agilent Technologies, USA). Then sequencing was carried out by the Illumina NovaSeq 6000 (Illumina, USA). The library construction and sequencing were executed at Shanghai Sinomics Corporation. Cuffdiff was used to evaluate DEGs. Log_2_| fold change| >1 and *P*< 0.05 were used to select DEGs.

### Gene Expression and Analysis of the TCGA Database

The RNA sequencing data (647 CRC cases vs. 51 normal cases) were downloaded from the TCGA database. The R software was used to analyze the data, and the “DESeq2” R package was used to acquire differently expressed lncRNAs. Log_2_| fold change| >1 and *P* value < 0.05 were used to pick statistically significant lncRNAs.

### Dual-Luciferase Analysis

The GZMB promoter region was constructed and inserted downstream of the luciferase reporter gene of the pGL3 primary vector, which contained a modified coding region for firefly luciferase. The TK vector, which included an area for renilla luciferase, was used as the control. All vectors were purchased from Biosune. Lipofectamine 2000 was used to transfect the reporter gene into DLD-1 cells. After transfection for 48 h, the Dual-Luciferase Reporter System Kit (Promega, USA, Cat#E1910) was used to test firefly and luciferase activities. The assay was repeated three times.

### Animal Experiments

For the *in vivo* metastasis experiments, 1 × 10^6^ DLD-1 cells stably transfected with sh-LINC02474 and the corresponding control were resuspended in 0.2 ml PBS and then injected into 14 BALB/c female nude mice by tail intravenous. The mice were 5-week-old and divided into two groups randomly (n=7 for each group). The mice were dissected after 50 days of injection, and lung tissues were isolated, fixed with formalin, and then stained with hematoxylin and eosin. All animal experiments were received and approved by the Institutional Animal Care and Use Committee of The Second Hospital, Cheeloo College of Medicine, Shandong University.

### Statistical Analysis

All data were presented as mean ± standard deviation (SD) from at least three independent experiments. The difference between the two groups was determined by two-tailed Student’s t-test, and the variance among multiple groups was analyzed by one-way analysis of variance (ANOVA). Overall survival (OS) curves were produced by the Kaplan-Meier method. The correlation between the expression of LINC02474 and clinical parameters was explored by the non-parametric Mann-Whitney test. Statistical analyses were performed with R software (version 3.5.2), SPSS software (version 19.0) (IBM, SPSS, Chicago, IL, USA) and GraphPad Prism 6 (GraphPad, La Jolla, CA, USA). A *P* value < 0.05 was considered statistically significant.

## Results

### Identification of Expression Profiles of LINC02474

**Figure 1:**
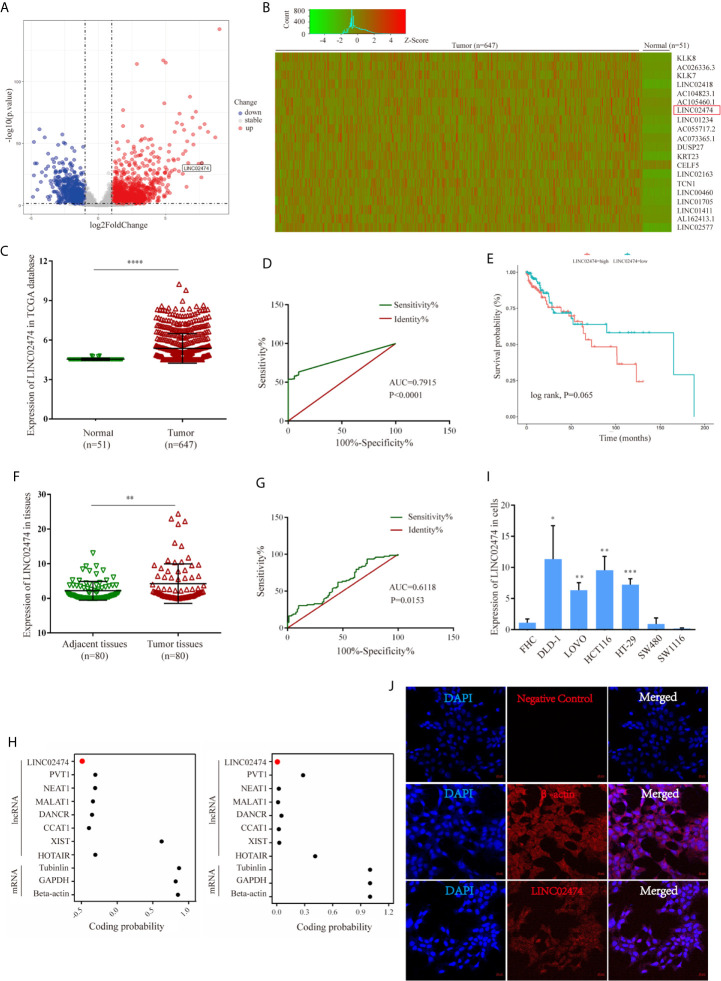
Identification of expression profile characteristics of LINC02474. **(A–E)** Expression profile characteristics of LINC02474 from TCGA data sets (TCGA-COAD and TCGA-READ). **(A)** Volcano plot of DEGs. Red dots (right) represent up-regulated genes; gray dots (middle) represent genes excluded from the threshold (|log_2_ fold change| >1, p < 0.05); and blue dots (left) represent down-regulated genes. **(B)** Heatmap of DEGs (http://shinyheatmap.com/). **(C)** Expression of LINC02474 in 51 normal tissues and 647 tumor tissues. **(D)** ROC curve of LINC02474 yielded an AUC value of 0.7915 (95% CI, 0.7495–0.8335) in distinguishing 647 CRC tissues from 51 normal tissues. **(E)** Survival analysis of LINC02474 from TCGA data sets. **(F)** Expression of LINC02474 in 80 pairs tissues from CRC patients. **(G)** ROC curve of LINC02474 yielded an AUC value of 0.6118 (95% CI, 0.5243–0.6994) in distinguishing tumor tissues from adjacent tissues in 80 CRC patients. **(H)** Coding probability prediction of LINC02474 (coding probability score<0 means no coding potential; coding probability score>0 means certain coding potential.) **(I)** Expression of LINC02474 in CRC cells (normalized to normal cell line, FHC.). **(J)** Subcellular localization of LINC02474 by RNA FISH. Red fluorescent probe: LINC02474 and β-actin (Cy3 labeled probes); blue fluorescent probe: DAPI. β-actin served as a positive control. Representative images (original magnification, ×200) are shown. Results are means ± SD. * represents p < 0.05; ** represents p < 0.01; *** represents p < 0.001; **** represents p < 0.0001.

We first accessed the raw RNA sequencing (RNA-seq) data of the CRC (TCGA-COAD and TCGA-READ) cohort study from The Cancer Genome Atlas (TCGA) database, including 51 normal tissues and 647 CRC tissues, to obtain the profiles of differentially expressed lncRNAs. Among the differentially expressed lncRNAs, we focused on a novel lncRNA, LINC02474, for further exploration. Analytical results from TCGA data suggested that LINC02474 was up-regulated in CRC tissues (**Figures 1A–C**). Moreover, receiver operating characteristic (ROC) analysis revealed that the expression of LINC02474 could be used to distinguish CRC tumor tissues from normal tissues (LINC02474: area under the curve [AUC] = 0.7915, 95% confidence interval [CI] = 0.7495–0.8335) (**Figure 1D**). Up-regulation of LINC02474 implied a poor survival although there was no statistical significance (**Figure 1E**). To validate the database results, we subsequently collected 80 pairs of tissue samples from CRC patients. By quantitative real-time polymerase chain reaction (qRT-PCR), LINC02474 was identified to be expressed at a higher level among human CRC tissues than adjacent normal tissues (*P*<0.05) (**Figure 1F**). ROC analysis confirmed that LINC02474 could also be used to distinguish tumor parts from adjacent regions in CRC patients (LINC02474: AUC = 0.6118, CI = 0.5243–0.6994) (**Figure 1G**). Besides, the clinical-pathological characterizations of CRC patients were analyzed by comparing with the expression of LINC02474; however, there was no significant correlation (**Supplementary Table 1**). Considering that recent studies have revealed that some lncRNAs can encode proteins, such as LINC00961 and lncRNA HOXB-AS3 (29–33), we also predicted the coding ability of LINC02474 in two web sites (http://lilab.research.bcm.edu/cpat/) (http://cnit.noncode.org/CNIT/) by definition of lncRNAs (34, 35). The site prediction results indeed revealed that LINC02474 did not have the protein-coding ability (**Figure 1H**). Moreover, we checked the endogenous expression of LINC02474 among human CRC cell lines, including DLD-1, LOVO, HCT116, SW1116, SW480, and HT-29. We found that the highest expression of LINC02474 was detected in DLD-1, LOVO, and HCT116 cells, while its lowest expression was found in SW480 and SW1116 cells (**Figure 1I**). Besides, the cellular localization of LINC02474 was identified by RNA fluorescence *in situ* hybridization (FISH) in DLD-1 cells. We found that the molecule was more enriched in the cytoplasm than the nucleus (**Figure 1J**). Taken together, a novel lncRNA, LINC02474, was detected and significantly up-regulated in CRC patients, indicating its potentials to be a biomarker in CRC diagnosis.

### Depletion of LINC02474 Represses Migration and Invasion but Accelerates the Apoptosis of CRC Cells

**Figure 2:**
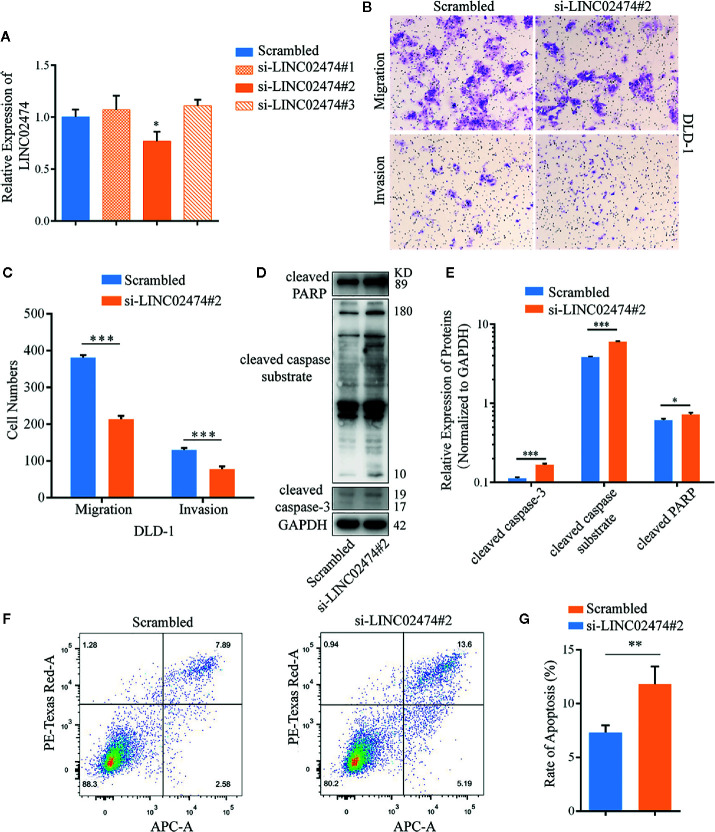
Depletion of LINC02474 with siRNAs may impede metastasis and promote apoptosis of CRC cells. **(A)** qRT-PCR results of LINC02474 expression by siRNAs. **(B, C)** Migration and invasion abilities of DLD-1 cells by transwell assay. Representative images (original magnification, ×100) are shown. **(D, E)** Expressions of apoptosis-related proteins (cleaved PARP, cleaved caspase substrate, and cleaved caspase-3) in DLD-1 cells after LINC02474 were depleted. **(F, G)** Apoptosis after LINC02474 depletion by flow cytometry. Results are means ± SD. * represents p < 0.05; ** represents p < 0.01; *** represents p < 0.001.

While we have shown that LINC02474 has a strong expression in CRC tissue, it remains uncertain if LINC02474 has any significant yet unexplained effect on CRC cells. Therefore, we depleted LINC02474 with short interfering RNAs (siRNAs) (**Figure 2A**). Compared with the negative control (scrambled), the migration and invasion abilities of DLD-1 cells treated by si-LINC02474#2 were affected (**Figures 2B, C**). Moreover, the expression of apoptosis-related proteins, including cleaved caspase-3, cleaved PARP, and cleaved caspase substrate, as well as apoptosis rate, were increased when LINC02474 was depleted, suggesting that LINC02474 also affected the apoptosis of CRC cells (**Figures 2D–G**). The results mentioned above implied that LINC02474 could mediate migration, invasion, and apoptosis of CRC cells.

**Figure 3:**
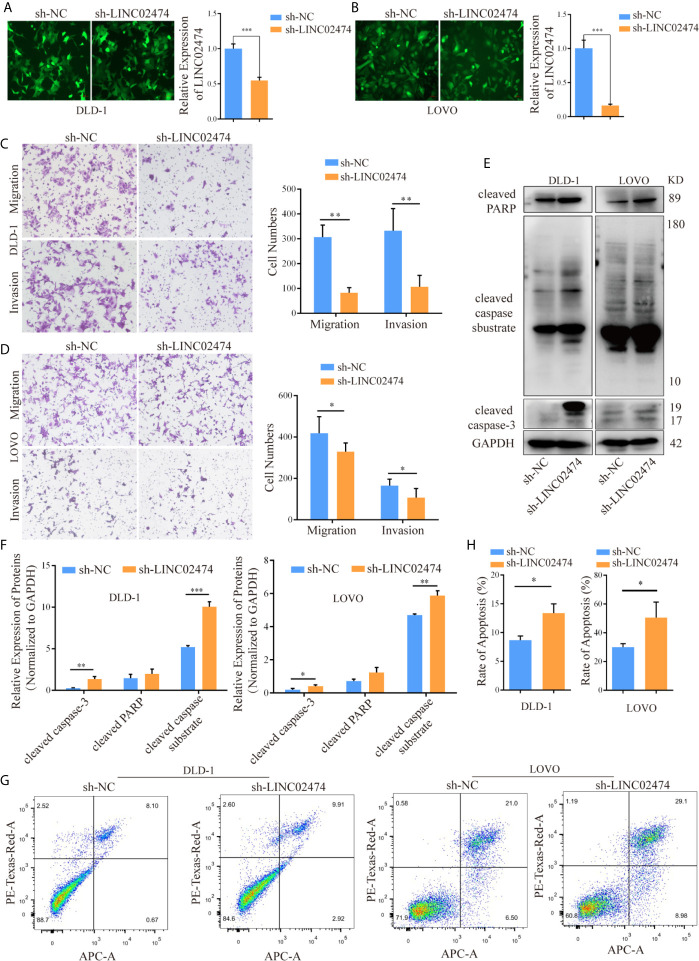
LINC02474, as an oncogene, promotes the abilities of migration and invasion but inhibits the apoptosis of CRC cells. **(A, B)** qRT-PCR results for knockdown efficiency of LINC02474 in DLD-1 and LOVO cells by shRNAs. Representative images (original magnification, ×200) are shown. **(C, D)** Migration and invasion abilities of DLD-1 and LOVO cells after LINC02474 was stably depleted. Representative images (original magnification, ×100) are shown. **(E, F)** Expressions of apoptosis-related proteins (cleaved PARP, cleaved caspase substrate, and cleaved caspase-3) in DLD-1 and LOVO cells after LINC02474 was stably depleted. **(G, H)** Apoptosis in DLD-1 and LOVO cells after LINC02474 was stably depleted by flow cytometry. Results are means ± SD. * represents p < 0.05; ** represents p < 0.01; *** represents p < 0.001.

To directly address whether LINC02474 affected metastasis and apoptosis of CRC cells, small hairpin RNA (shRNA) plasmid vectors were designed and transferred into HEK293T cells. Then supernatants of HEK293T cells containing viral particles were collected and incubated with CRC cells, such as DLD-1, LOVO, and HCT116 cells, for 48 h to generate cell lines with stable depletion of LINC02474 was stably depleted (**Figures 3A, B** and **Supplementary Figure 1A**). In line with the previous data, the transwell assay revealed that the three cell lines’ migration and invasion abilities in the sh-LINC02474 group were reduced compared with the negative control group (**Figures 3C, D** and **Supplementary Figure 1B**). The apoptosis was increased after LINC02474 was stably depleted, consistent with our previous findings (**Figures 3E–H** and **Supplementary Figures 1C, D**).

**Figure 4:**
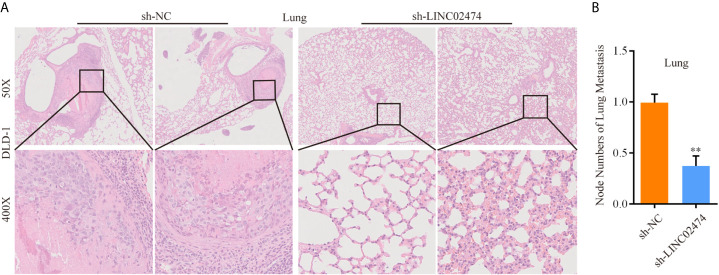
LINC02474 promotes tumor metastasis *in vivo*. **(A)** Representative microscopic images of metastatic pulmonary lesions in two group nude mice (n = 7 for each group). Representative images (original magnification, ×50 [up] and ×400 [down]) are shown. **(B)** The number of metastatic nodules in the lung were counted. ** represents p < 0.01.

Similarly, we synthesized the exogenous LINC02474 plasmid vector and established LINC02474-overexpressing cell lines using SW1116 and SW480 cells (**Supplementary Figures 2A, B**). As expected, the invasion and migration abilities of LINC02474-overexpressing cells were significantly increased in comparison with those transfected by pcDNA3.1 empty vector (**Supplementary Figures 2C–F**). The apoptosis rate was decreased when LINC02474 was overexpressed in SW1116 and SW480 cells (**Supplementary Figures 2G–J**). Besides, to investigate whether LINC02474 also promoted CRC metastasis *in vivo*, we intravenously injected LINC02474-depleted DLD-1 cells into nude mice. In the metastatic nude mouse model by tail vein, sh-LINC02474 significantly prevented the formation of pulmonary metastatic nodules compared the control group after 50 days (**Figures 4A, B**). Altogether, the results as mentioned earlier suggested that LINC02474 was an oncogenic lncRNA, and promoted tumor metastasis both *in vivo* and *in vitro* but inhibited CRC cells’ apoptosis.

### Identification of Target Genes of LINC02474 by RNA-seq

This study showed that depletion of LINC02474 could damage metastasis but facilitate apoptosis of CRC cells. However, the underlying mechanism remained unknown. Therefore, we performed RNA-seq in DLD-1 cells by depletion of LINC02474 or not.

**Figure 5:**
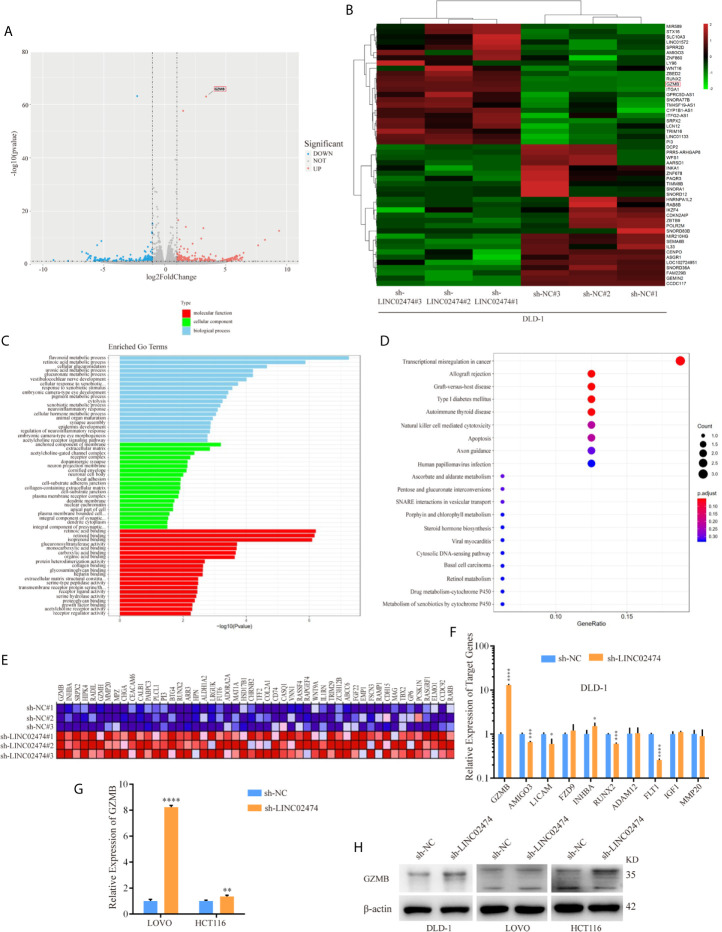
Identification of target genes for LINC02474 with RNA-seq. **(A)** Volcano plot and **(B)** Heatmap after LINC02474 depletion in DLD-1 cells. **(C)** GO, **(D)** KEGG, and **(E)** GSEA gene markers after LINC02474 depletion in DLD-1 cells. **(F, G)** qRT-PCR results for expression identification of selected genes in DLD-1, LOVO, and HCT116 cells. **(H)** Protein expression of GZMB in DLD-1, LOVO, and HCT116 cells. Results are means ± SD. * represents p < 0.05 ; ** represents p < 0.01; *** represents p < 0.001; **** represents p < 0.0001.

The RNA-seq data showed that hundreds of differentially expressed genes (DEGs) were identified between the LINC02474 depletion group and the negative control group (**Figures 5A, B**). Gene Ontology (GO) analysis showed that the most significantly overrepresented cellular component included an anchored part of the membrane and extracellular matrix (**Figure 5C**). Interestingly, the Kyoto Encyclopedia of Genes and Genomes (KEGG) analysis showed that most genes were enriched in the pathway named transcriptional misregulation in cancer (**Figure 5D**). The Gene Set Enrichment Analysis (GSEA) also revealed the gene markers between the sh-LINC02474 group and the control, and GZMB was ranked first (**Figure 5E**). Then genes involved in the transcriptional misregulation pathway, including GZMB, AMIGO3, L1CAM, FZD9, INHBA, RUNX2, ADAM12, FLT1, IGF1, and MMP20, were selected from DEG profiles and verified by qRT-PCR in DLD-1 cells (**Figure 5F**). GZMB was found to be significantly up-regulated among selected genes, which was consistent with our sequencing results. The other two CRC cell lines, LOVO, and HCT116, were examined for further identification, and found similar up-regulation was found in the sh-LINC02474 group. (**Figure 5G**). Besides, the protein expression of GZMB was also increased when LINC02474 was reduced (**Figure 5H**). Taken together, the data mentioned above implied that LINC02474 could suppress the expression of GZMB.

### Influence of LINC02474 on CRC Cells Can Be Mediated by Restricting GZMB

**Figure 6:**
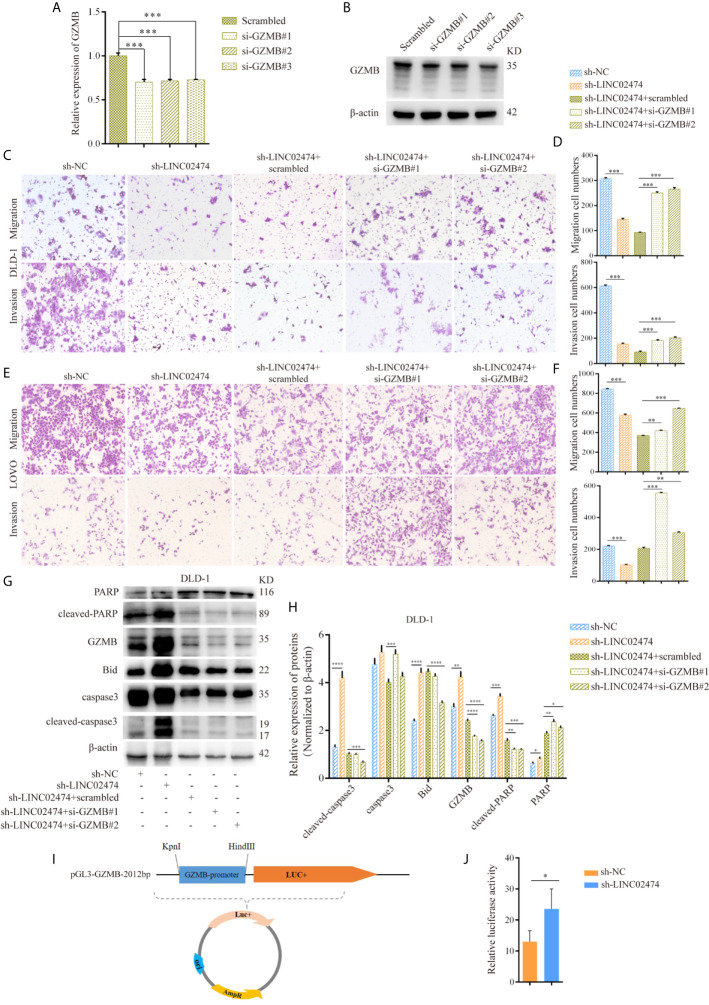
The influence of LINC02474 on CRC cells can be mediated by restricting transcriptional regulation of GZMB. **(A)** qRT-PCR and **(B)** Western blotting analysis for the expression of GZMB by siRNAs. **(C–F)** Migration and invasion after GZMB depletion in DLD-1 and LOVO cells, in which LINC02474 was stably depleted. Representative images (original magnification, ×100) are shown. **(G, H)** Expressions of apoptosis-related proteins and GZMB after depleting GZMB in DLD-1 cells, in which LINC02474 was down-regulated. **(I)** Dual-luciferase reporter gene vector of GZMB gene promoter. **(J)** Relative luciferase activity of GZMB gene promoter after LINC02474 was depleted. Results are means ± SD. * represents p < 0.05; ** represents p < 0.01; *** represents p < 0.001; **** represents p < 0.0001.

Based on the above experiments, we hypothesized that the function of LINC02474 could be regulated by the expression of GZMB. Therefore, we designed siRNAs targeting GZMB and transferred them into LINC02474-depleted DLD-1 cells, and the expression of GZMB both in mRNA and protein levels was assessed (**Figures 6A, B**). Compared with the negative control, we found that the impaired migration and invasion abilities could be restored by siRNAs of GZMB (**Figures 6C–F** and **Supplementary Figures 3A, B**), implying that GZMB was involved in the metastatic process of CRC cells. On the other hand, the expressions of apoptosis-related proteins, including cleaved PARP and cleaved caspase-3, were reduced when GZMB was depleted. Besides, we found that the expression of another related protein, Bid, was decreased, which was the substrate of GZMB and functioned as a proteolytic enzyme (**Figures 6G, H**). Accordingly, some previous studies have shown that GZMB can cleave Bid and caspase-3 and induce apoptosis in cells. These results supported that LINC02474 exerted an influence on metastasis and apoptosis of CRC cells mainly by suppressing the expression of GZMB.

Due to the effects of LINC02474 on GZMB, we further tested the mechanism underlying the GZMB-regulated inhibition. Since we showed that LINC02474 could suppress the expression of GZMB at the mRNA level, we assessed whether LINC02474 could impede the stability of GZMB mRNA. The LINC02474-depleted DLD-1 cells were treated with actinomycin D (ActD) for different durations, and the mRNA stability of GZMB was analyzed by qRT-PCR. Surprisingly, there were no effects of LINC0474 on stability (**Supplementary Figure 3C**). Next, to explore whether LINC02474 hindered the transcriptional regulation of GZMB, we designed a dual-luciferase reporter system, which included the promotor region of GZMB (**Figure 6I**). After the plasmid vector was transferred into DLD-1 cells, the fluorescence intensity was more vigorous in LINC02474-depleted cells (**Figure 6J**). These findings provided direct evidence that LINC02474 could contribute to the blocking of the transcriptional regulation of GZMB.

Collectively, the results mentioned above indicated that LINC02474 could affect metastasis and apoptosis in CRC cells by suppressing the transcriptional regulation of GZMB.

## Discussion

The previous studies have mainly focused on the roles of protein-coding genes. However, many ncRNAs have been recently found to influence on the cellular process and progression of human diseases significantly. In particular, the impact of lncRNAs on tumor progression has been a hot research spot (36–39). For instance, lncRNA SATB2-AS1 has specifically low CRC expression, and its down-regulation is related to poor survival (40). Moreover, lncRNA CGLL1, a functionally, mechanistically, and clinically active oncogene, can promote tumor carcinogenesis and glucose metabolism in CRC (41). Similarly, a novel lncRNA, named LINC02474, was identified to be localized on chromosome 1 by employing lncRNA expression profile data downloaded from public databases. Besides, we hypothesized that up-regulated LINC02474 was associated with a poor prognosis, and it could be used as a prognostic biomarker.

Depletion of LINC02474 could damage metastasis *in vitro* and *in vivo* but promote apoptosis of CRC cells. Subsequently, RNA-seq data showed that depletion of LINC02474 might affect the expressions of cancer-related genes, such as GZMB. Furthermore, LINC02474 could inhibit the expression of GZMB not only at the mRNA level but also at the protein aspect. To further identify the inhibition of LINC02474, we knocked down GZMB in LINC02474-depleted cells with siRNAs and found that the impaired migration, invasion, and apoptosis abilities caused by LINC02474 depletion could be restored by down-regulating GZMB. Therefore, we speculated that LINC02474 exerted its effect mainly by inhibiting GZMB. Finally, with more in-depth mechanism exploration, we found that LINC02474 might affect the transcription of GZMB to regulate its expression at the mRNA level, not through RNA stability.

Previous reports have shown that GZMB is usually expressed in immune cells, such as NK cells and CTLs, and it performs cell killing ability in the tumor microenvironment (42–44). However, only very few studies have focused on the role of GZMB in cancer cells, even less in CRC cells. It has been found that low expression of GZMB is related to early metastasis, indicating invasion of blood vessels and nerves (26). Furthermore, a recent study has proved that the expression of GZMB at the mRNA level is lower in early metastatic CRC (45). The present study found that GZMB could be expressed in CRC cells, and LINC02474 might inhibit its expression through transcriptional regulation.

Taken together, we identified a novel lncRNA, LINC02474, which could affect migration and invasion as well as apoptosis by inhibiting the expression of GZMB in CRC. Moreover, the high expression of LINC02474 might be associated with a poor prognosis. Therefore, LINC02474 could potentially be potential biomarker for CRC prognosis.

## Data Availability Statement

The raw and processed data of High-throughput Sequencing (Next Generation Sequencing) can been accessed with accession number GSE169722 (https://www.ncbi.nlm.nih.gov/geo/query/acc.cgi?acc=GSE169722) in the Gene Expression Omnibus (GEO) database.

## Ethics Statement

The studies involving human participants were reviewed and approved by the Committee for Ethical Review of Research involving Human Subjects of the Second Hospital, Cheeloo College of Medicine, Shandong University. The patients/participants provided their written informed consent to participate in this study. The animal study was reviewed and approved by the Institutional Animal Care and Use Committee of The Second Hospital, Cheeloo College of Medicine, Shandong University.

## Author Contributions

CW and LD conceived and designed this study. TD and QG performed the experiments, conducted the data analysis, and prepared figures and tables. YZ, JG, JL, LW, PL, and YW provided technical support and revised the article. CW, LD, TD, and QG wrote the manuscript. All authors have read and approved the final version of the manuscript.

## Funding

This research was supported by a grant from the National Natural Science Foundation of China (81972007 and 82002228), the National Key Research and Development Program of China (2018YFC0114700), the Key Research and Development Program of Shandong Province (2019GHZ003, 2018YFJH0505 and 2019GSF108206), the Natural Science Foundation of Shandong Province (ZR201910250056), and the Fundamental Research Funds of Shandong University (2082018JC002).

## Conflict of Interest

The authors declare that the research was conducted in the absence of any commercial or financial relationships that could be construed as a potential conflict of interest.

## Supplementary Material

Supplementary Figure 1 LINC02474, as an oncogene, promotes the migration, and invasion but inhibits the apoptosis of CRC cells. **(A)** qRT-PCR results for knockdown efficiency of LINC02474 in HCT116 by shRNAs. Representative images (original magnification, ×200) are shown. **(B)** Migration and invasion abilities of HCT116 cells after LINC02474 was stably depleted. Representative images (original magnification, ×100) are shown. **(C)** Apoptosis in HCT116 cells after LINC02474 was stably depleted. **(D)** Expressions of apoptosis-related proteins (cleaved PARP, cleaved caspase substrate, and cleaved caspase-3) in HCT116 cells after LINC02474 being stably depleted. Results are means ± SD. ** represents p<0.01; *** represents p<0.001.

Supplementary Figure 2 Overexpression of LINC02474 promotes the migration and invasion but reduces the apoptosis of CRC cells. **(A, B)** qRT-PCR results for overexpression efficiency of LINC02474 in SW1116 and SW480 cells. Representative images (original magnification, ×200) are shown. **(C–F)** Migration and invasion abilities of SW1116 and SW480 cells after LINC02474 was stably overexpressed. Representative images (original magnification, ×100) are shown. **(G–J)** Apoptosis in SW1116 and SW480 cells after LINC02474 was stably overexpressed. Results are means ± SD. ** represents p<0.01; **** represents p<0.0001.

Supplementary Figure 3 The influence of LINC02474 on CRC cells is not associated with the RNA stability of GZMB. **(A, B)** Migration and invasion after GZMB knockdown in HCT116 cells, in which LINC02474 was stably depleted. Representative images (original magnification, ×100) are shown. **(C)** RNA stability of GZMB by ActD when LINC02474 was depleted, and c-myc served as a positive control. Results are means ± SD. ** represents p<0.01; *** represents p<0.001.
