# Supplementary figures and images for: Long Non-coding RNA LINC02474 Affects Metastasis and Apoptosis of Colorectal Cancer by Inhibiting the Expression of GZMB
Source: Front Oncol. 2021 Apr 9;11:651796. doi: 10.3389/fonc.2021.651796 (PMC8063044; doi:10.3389/fonc.2021.651796)

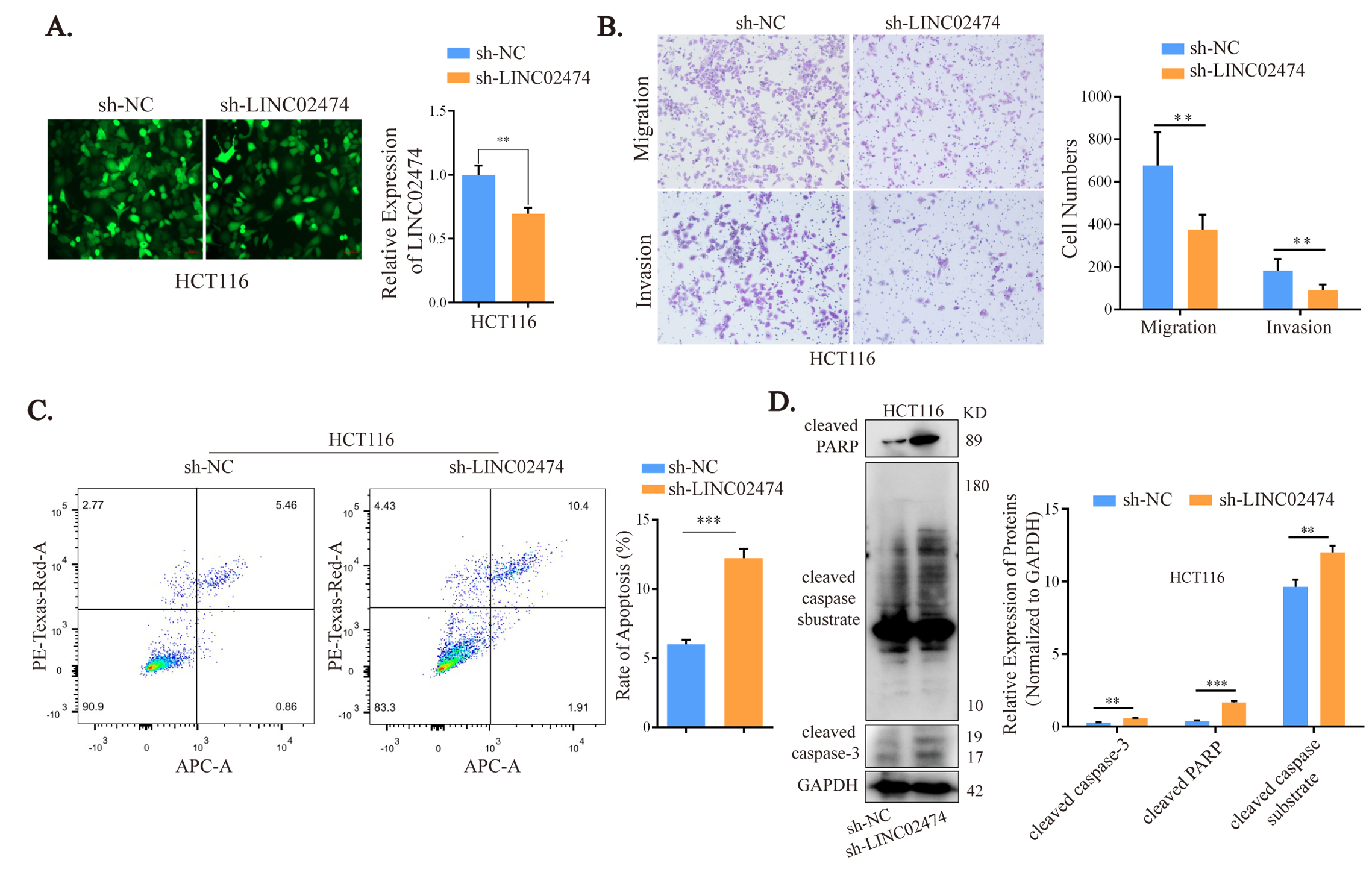

Supplement: Supplementary Figure 1 — LINC02474, as an oncogene, promotes the migration, and invasion but inhibits the apoptosis of CRC cells. (A) qRT-PCR results for knockdown efficiency of LINC02474 in HCT116 by shRNAs. Representative images (original magnification, ×200) are shown. (B) Migration and invasion abilities of HCT116 cells after LINC02474 was stably depleted. Representative images (original magnification, ×100) are shown. (C) Apoptosis in HCT116 cells after LINC02474 was stably depleted. (D) Expressions of apoptosis-related proteins (cleaved PARP, cleaved caspase substrate, and cleaved caspase-3) in HCT116 cells after LINC02474 being stably depleted. Results are means ± SD. ** represents p<0.01; *** represents p<0.001. [file Image_1.tif]

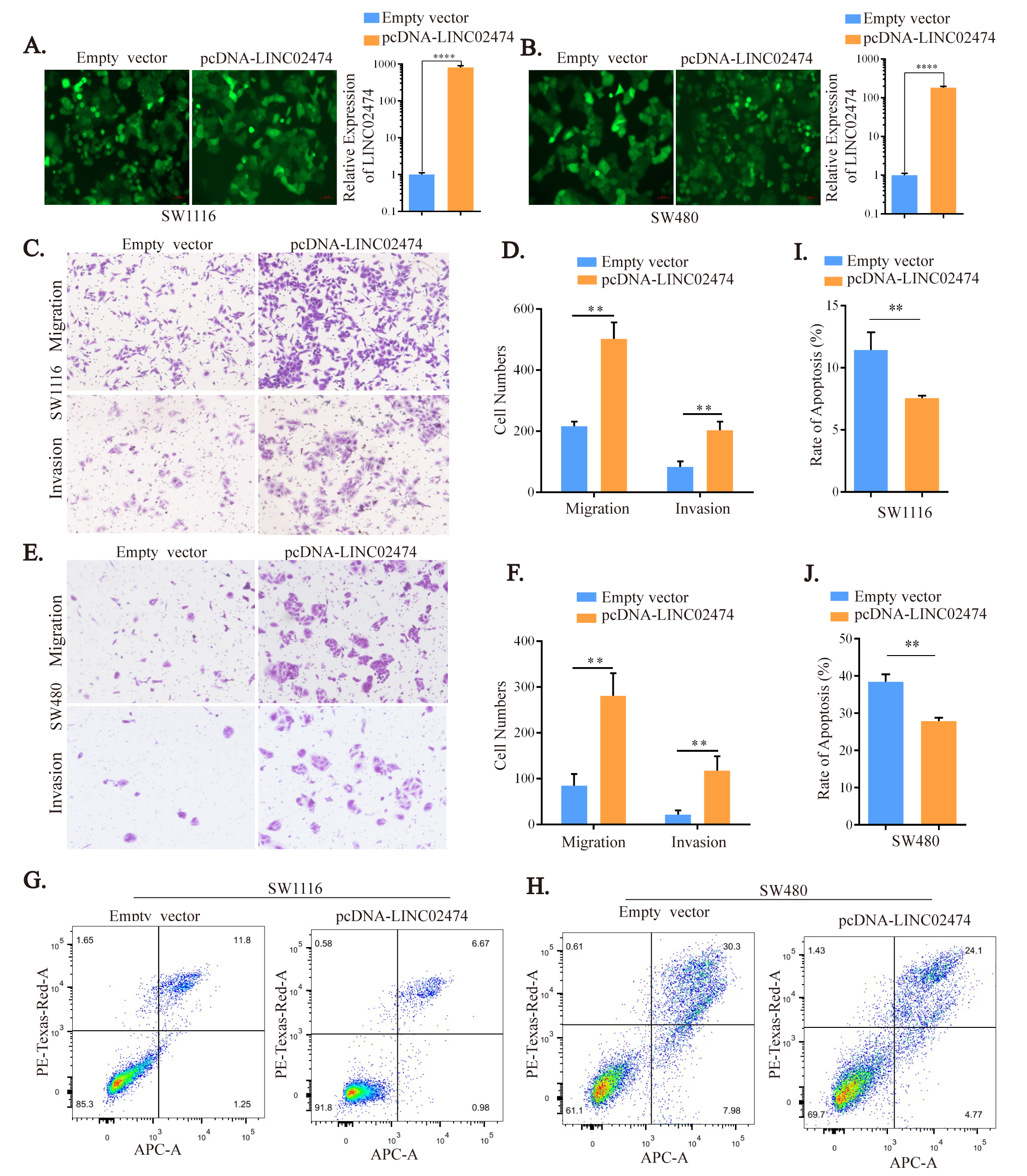

Supplement: Supplementary Figure 2 — Overexpression of LINC02474 promotes the migration and invasion but reduces the apoptosis of CRC cells. (A, B) qRT-PCR results for overexpression efficiency of LINC02474 in SW1116 and SW480 cells. Representative images (original magnification, ×200) are shown. (C–F) Migration and invasion abilities of SW1116 and SW480 cells after LINC02474 was stably overexpressed. Representative images (original magnification, ×100) are shown. (G–J) Apoptosis in SW1116 and SW480 cells after LINC02474 was stably overexpressed. Results are means ± SD. ** represents p<0.01; **** represents p<0.0001. [file Image_2.tif]

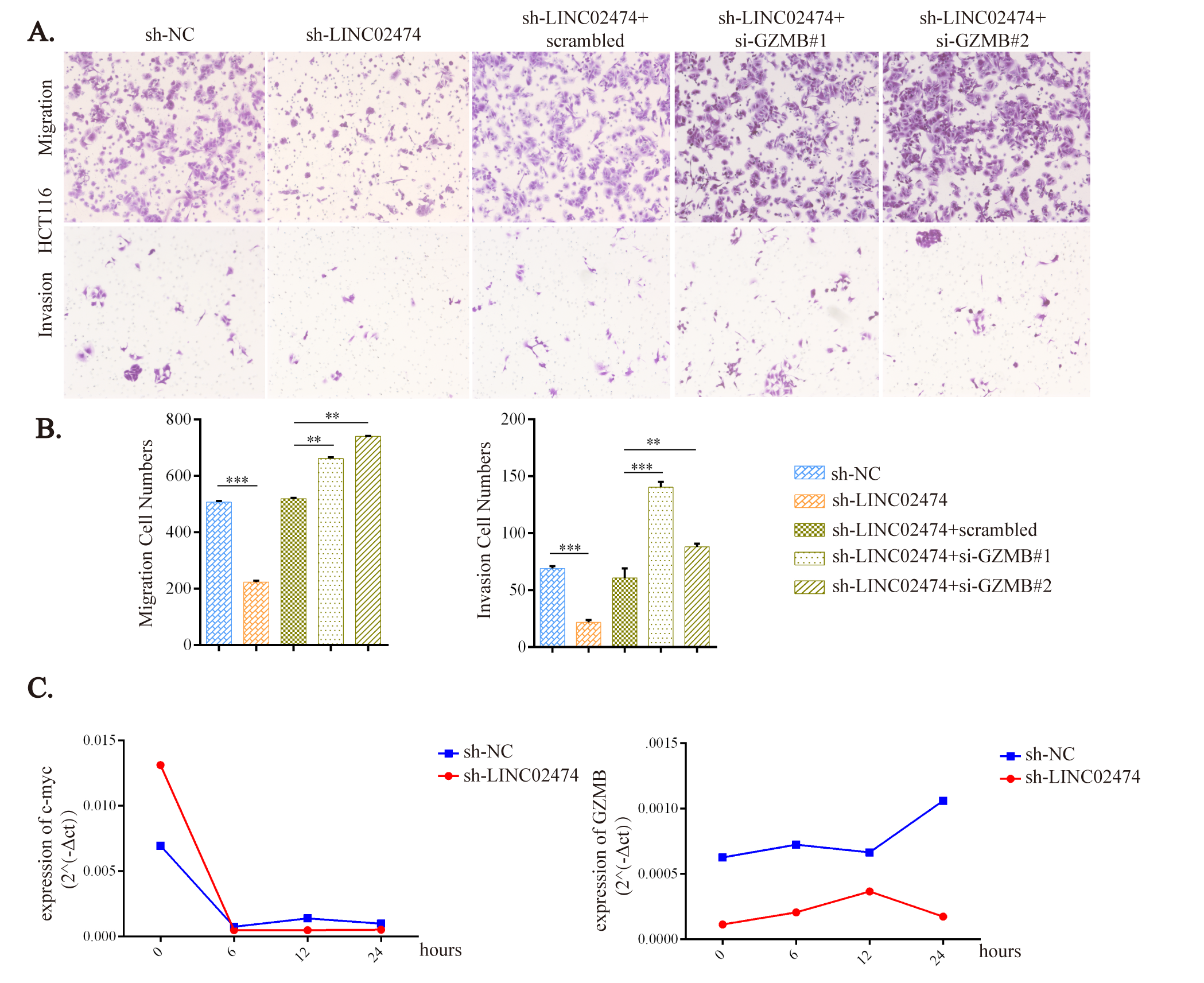

Supplement: Supplementary Figure 3 — The influence of LINC02474 on CRC cells is not associated with the RNA stability of GZMB. (A, B) Migration and invasion after GZMB knockdown in HCT116 cells, in which LINC02474 was stably depleted. Representative images (original magnification, ×100) are shown. (C) RNA stability of GZMB by ActD when LINC02474 was depleted, and c-myc served as a positive control. Results are means ± SD. ** represents p<0.01; *** represents p<0.001. [file Image_3.tif]
