# Supplementary material for: Long Non-coding RNA LINC02474 Affects Metastasis and Apoptosis of Colorectal Cancer by Inhibiting the Expression of GZMB
Source: Front Oncol. 2021 Apr 9;11:651796. doi: 10.3389/fonc.2021.651796 (PMC8063044; doi:10.3389/fonc.2021.651796)
Supplement: Supplementary file 4 [file Table_1.docx]

**Supplementary Table 1. The correlation between the expression of LINC02474 and clinical-pathological characterizations of CRC patients.**

| **Parameters** | **Total case** | **LINC02474** | **P value** |
| --- | --- | --- | --- |
| **Age(years)** |  |  | 0.9713 |
| ＜62 | 37 | 1.558（0.3831-5.932） |  |
| ≥62 | 43 | 1.579（0.4118-9.547） |  |
| **Sex**  Male |  |  | 0.3590 |
|  | 47 | 1.558（0.2176-5.098） |  |
| Female | 33 | 1.597（0.5899-10.69） |  |
| **Tumor size** |  |  | 0.4818 |
| ≤5cm | 56 | 1.506（0.4715-15.67） |  |
| ＞5cm | 18 | 1.675（0.1630-2.838） |  |
| NA | 6 | NA |  |
| **Lymph node metastasis** |  |  | 0.8021 |
| Negative | 34 | 1.228（0.3916-10.36） |  |
| Positive | 40 | 1.706（0.4515-5.899） |  |
| NA | 6 | NA |  |
| **Distant metastasis** |  |  | 0.4141 |
| No | 59 | 1.659（0.4863-11.84） |  |
| Yes | 15 | 1.591（0.1303-5.098） |  |
| NA | 6 | NA |  |
| **TNM stage** |  |  | 0.5072 |
| Ⅰ  Ⅱ | 5 | 3.138（0.3310-11.14） |  |
|  | 22 | 0.7834（0.4326-29.97） |  |
| Ⅲ | 32 | 1.706（0.7041-5.899） |  |
| Ⅳ | 15 | 1.591（0.1303-5.098） |  |
| NA | 6 | NA |  |

Note: NA represents no value.
